# Influence of lattice defects on the ferromagnetic resonance behaviour of 2D magnonic crystals

**DOI:** 10.1038/srep22004

**Published:** 2016-02-25

**Authors:** Alessandra Manzin, Gabriele Barrera, Federica Celegato, Marco Coïsson, Paola Tiberto

**Affiliations:** 1 Istituto Nazionale di Ricerca Metrologica (INRIM), Torino, Italy; 2 Università degli Studi di Torino, Dipartimento di Chimica, Torino, Italy

## Abstract

This paper studies, from a modelling point of view, the influence of randomly distributed lattice defects (non-patterned areas and variable hole size) on the ferromagnetic resonance behaviour and spin wave mode profiles of 2D magnonic crystals based on Ni_80_Fe_20_ antidot arrays with hexagonal lattice. A reference sample is first defined via the comparison of experimental and simulated hysteresis loops and magnetoresistive curves of patterned films, prepared by self-assembly of polystyrene nanospheres. Second, a parametric analysis of the dynamic response is performed, investigating how edge, quasi-uniform and localized modes are affected by alterations of the lattice geometry and bias field amplitude. Finally, some results about the possible use of magnetic antidot arrays in frequency-based sensors for magnetic bead detection are presented, highlighting the need for an accurate control of microstructural features.

The recent advances in nanofabrication techniques have given a boost to the study of artificially patterned magnetic films for future applications in high density magnetic storage, sensor technology and magneto-logic devices. Attention has been focused on magnetic antidot arrays (magnetic thin films with periodic non-magnetic inclusions or embedded holes), whose hysteresis, anisotropy, magnetization reversal and magnetotransport behaviour can be engineered by properly varying the hole arrangement^1^,^2^,^3^,^4^. The introduction of local shape anisotropies makes these systems interesting also for magnetization dynamics and spin wave propagation properties, which can be exploited for the design of magnonic-crystal waveguides, spin wave emitters, nanoscale microwave filters and frequency-based magnetic nanoparticle detectors^5^,^6^,^7^,^8^,^9^. Their ferromagnetic resonance (FMR) behaviour is strongly influenced by the lattice geometrical parameters and can be artificially tuned by modifying the shape and size of the non-magnetic inclusions, as well as their arrangement and packing fraction^10^,^11^,^12^,^13^,^14^. In such scenario, the accurate control of geometrical properties at the microscopic level is of fundamental importance to avoid alterations of the expected magnetization dynamics and resonance modes.

Electron beam lithography (EBL) enables to obtain magnetic nanopatterns with well-defined lattice features, at the cost of low speed and small covering area due to the sequential nature of the writing process^15^. Conversely, self-assembly techniques can guarantee low-cost fabrication and large patterning area, but the achievement of ordered geometrical configurations is a very complex task. Different methods have been employed to obtain self-assembled magnetic antidot arrays, based on the use of block copolymer templates^16^,^17^,^18^,^19^, lyotropic liquid crystals solutions^20^, anodized alumina membranes^21^,^22^,^23^,^24^ and close packed arrays of polystyrene spheres^25^,^26^,^27^. Despite the hexagonal short-range hole order, the antidot arrays obtained by self-assembling techniques may not sustain long-range hole ordering on the whole sample. Disorder in the morphological structure can be ascribed to hole-ordered regions forming lattice domains aligned towards randomly distributed directions, and to more local defects, such as non-patterned areas and variable hole size^15^.

Modifications of the morphological properties of the patterned film have a direct impact on the static behaviour and magnetization reversal mechanism, as observed in cobalt antidot arrays fabricated on anodized alumina membranes^24^ and in EBL Ni_80_Fe_20_ antidot arrays with engineered defects (missing holes)^28^. At the same time, they can influence the dynamic behaviour, leading to a variation in the strongly localized modes arising at the hole edges and thus in the FMR spectra. In a preliminary modelling study about the role of defects (filled holes and chains of filled holes in the lattice), it was concluded that they do not affect the magnonic spectra in a significant way^29^. However, their effects on the FMR modes (strongly dependent on bias field and excitation conditions) and their implications on device applications are still to be understood. Recently, a strong contribution from lattice irregularities was observed in Ni_80_Fe_20_ antidot arrays on alumina nanoporous membranes: even for quite regular hexagonal arrangement of the holes, the angular dependence of the FMR spectra does not exhibit the expected six-fold symmetry^30^.

In the present work, we investigate from a modelling point of view the influence of randomly distributed lattice defects (introducing both non-patterned areas and variable hole size) on the FMR behaviour and spin wave mode profiles of 2D Ni_80_Fe_20_ antidot arrays with hexagonal lattice. In order to have a realistic distribution of disorder, we simulate large-scale samples (with ∼6–8 μm size) by means of a GPU-based micromagnetic solver, which combines a Fast Multipole method for the magnetostatic field calculation to a finite difference technique for the exchange field evaluation on non-structured meshes^31^,^32^,^33^,^34^.

First, we define a reference sample for the numerical study starting from the comparison between experimental and simulated static hysteresis loops and magnetoresistance curves of Ni_80_Fe_20_ antidot arrays prepared by self-assembly of polystyrene nanospheres, with average hole diameter of 330 nm and centre-to-centre distance of about 500 nm. Second, we perform a parametric analysis of their dynamic behaviour focusing on the role of defect density (percentage of filled holes), local variations in the hole diameter and dc bias field amplitude on FMR frequencies and spin wave mode profiles (i.e., spatial distributions of mode spin precession amplitude). Finally, some hints are provided about the possible use of magnetic antidot arrays as active elements of frequency-based sensors for magnetic nano/microparticle detection, putting in evidence the need for a very accurate control of the lattice properties.

## results

### Definition of reference sample and static properties

**Figure 1:**
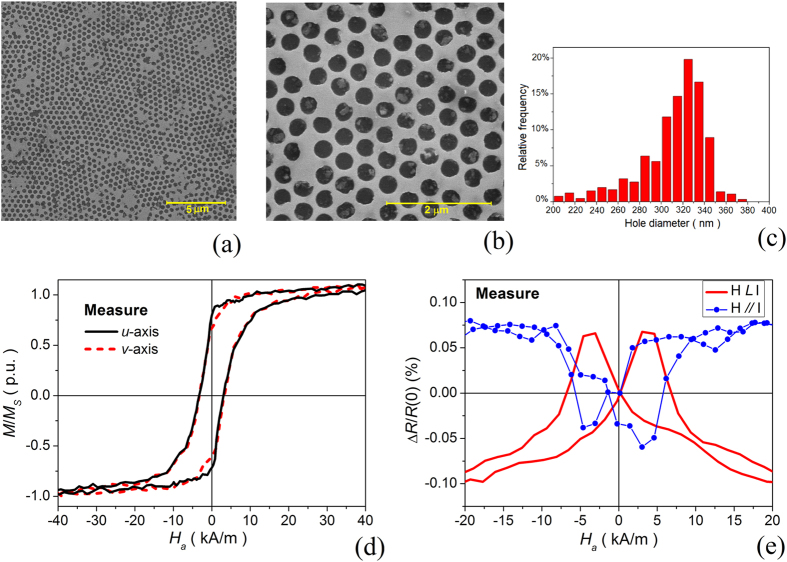
(**a**) Scanning electron microscopy (SEM) image of the considered Ni_80_Fe_20_ antidot array prepared by self-assembling of polystyrene nanospheres and (**b**) magnification. (**c**) Distribution of hole diameters. (**d**) Room-temperature hysteresis loops measured with the applied field in the film plane, oriented along two arbitrary orthogonal directions (labelled as *u*- and *v*-axis). (**e**) Room-temperature AMR curves measured for longitudinal (parallel) and transverse (perpendicular) configurations.

The reference sample for the parametric analysis is defined by searching for a virtual model that approximately mimics disorder distribution in antidot arrays, leading to a good reproduction of experimental hysterestic and magnetoresistive behaviour. To this aim, we consider Ni_80_Fe_20_ patterned thin films fabricated via a photolithographic technique that uses a lithography mask based on polystyrene nanosphere self-assembling. The resulting antidot array, with thickness of 30 nm, has an average hole diameter of 330 nm (Fig. 1c) and a centre-to-centre hole distance of about 500 nm. As can be observed in the scanning electron microscopy (SEM) images of Fig. 1a,b, the nanostructured film is characterized by hexagonal short-range hole order (on the micrometer scale), with point defects, dislocations, non-patterned areas and lattice domain boundaries on a larger scale. The average percentage of filled holes is ∼7.5%.

**Figure 2:**
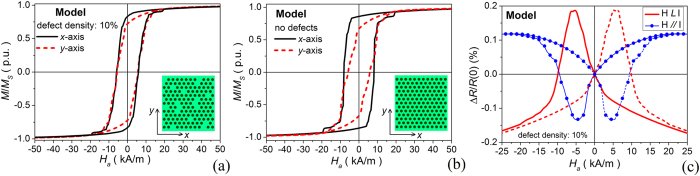
(**a**) Calculated hysteresis loops (along *x*- and *y*-axis directions) for the disordered antidot array with 10% of filled holes, spatially-distributed as depicted in the inserted scheme. (**b**) Hysteresis loops computed for the corresponding ordered structure. (**c**) AMR curves (for both longitudinal and transverse configurations) calculated for the disordered antidot array with 10% of filled holes.

Figure 1d shows the corresponding room-temperature hysteresis loops, measured by means of a vibrating sample magnetometer (VSM), with the field applied in the film plane along two arbitrary orthogonal directions (labelled as *u*- and *v*-axis). Due to lattice misalignment, it is not possible to identify well-defined crystallographic orientations for the whole sample. Very similar loop shapes, remanent magnetization values and coercive fields are obtained by applying the external field along any directions in the film plane. This result demonstrates how disorder impacts in a strong way on the pattern anisotropy properties with the disappearance of a preferred lattice orientation. It is worth noting that in ordered hexagonal antidot arrays with similar geometrical features a six-fold anisotropy was theoretically and experimentally observed, with easy and hard axes alternating every 30 °. In particular, the easy axes are aligned along the directions where the non-magnetic inclusions are closest to each other^1^,^35^. By referring to the scheme in Fig. 2b, these correspond to *x*-axis and geometrically equivalent directions, shifted in multiples of 60 °.

The lack of a preferred orientation is also demonstrated by the room-temperature magnetoresistance curves, measured for both longitudinal (applied field parallel to electrical current) and transverse (applied field perpendicular to electrical current) configurations (Fig. 1e). The system tendency to isotropy leads to anisotropic magnetoresistance (AMR) signals with similar amplitude for the two considered experimental conditions.

A good numerical reconstruction of the experimental static hysteresis loops and magnetoresistive curves can be obtained by considering a 6 μm size film with a percentage of randomly distributed filled holes of 10%, independently of their position (see Fig. 2a). In comparison to the experimental sample, a higher percentage of filled holes is introduced to compensate for the disregarding of lattice misalignment, which contributes to the complete disappearance of preferred lattice orientations at the macroscale. The introduction of non-patterned areas causes a strong weakening of the six-fold anisotropy, as can be seen from the comparison with the hysteresis loops computed for an ordered array, where a broad variation in coercive field values and loop shapes is found for the two considered field directions. The strongly reduced anisotropy can be also deduced from the simulated AMR curves reported in Fig. 2c.

**Figure 3:**
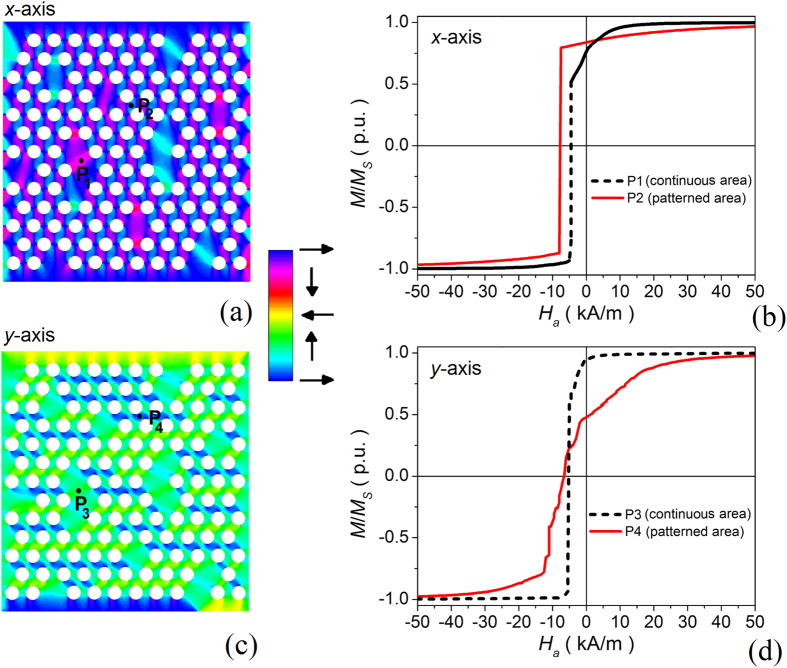
(**a**) Magnetic domain configuration calculated at remanence for the *x*-axis loop. (**b**) Localized reversal curves computed in points, indicated in (**a**), belonging to patterned and continuous regions of the antidot array (field applied along *x*-axis). (**c**) Magnetic domain configuration calculated at remanence for the *y*-axis loop. (**d**) Localized reversal curves computed in points, indicated in (**c**), belonging to patterned and continuous regions of the antidot array (field applied along *y*-axis). The colour scale associated with the magnetization maps identifies the magnetization direction with respect to *x*-axis.

The behaviour of the system can be more easily understood by analyzing the calculated spatial distributions of the magnetization at remanence state (shown in Fig. 3a,c for the two considered directions of the applied field). The presence of non-patterned regions breaks periodicity, perturbing the leaf-type configuration of magnetic domains induced by the holes. Moreover, magnetization rotation is not synchronous in the patterned and continuous areas. In particular, for the remanence state associated with the *x*-axis loop, the non-patterned regions are characterized by a preceding magnetization switching, as demonstrated by Fig. 3b, which reports the localized reversal curves calculated in specific points of the antidot array (indicated in Fig. 3a). This result explains the reduction in coercive field and remanent magnetization due to the introduction of disorder, when the field is applied along the easy axis direction for the ordered structure. For the remanence state associated with the *y*-axis loop (Fig. 3c), the leaf-type configuration in the patterned regions is rotated of 30° with respect to *y*-axis (being the hard direction in the absence of disorder). In the continuous areas the rotation of the magnetization is initially delayed (Fig. 3d), with a consequent increase in the remanent magnetization and coercive field with respect to the case of ordered antidot array.

### Ferromagnetic resonance behaviour

The influence of microstructural defects on FMR behaviour and spin wave mode profiles is investigated considering 8 μm size films, with thickness of 10 nm. Following the conclusions of the previous analysis, an antidot array with 10% of filled holes is assumed as a reference, fixing the hole diameter to 330 nm and the centre-to-centre hole distance to 500 nm. Different aspects are analyzed, such as the role of defect density (percentage of filled holes), local variations in the hole size, and amplitude of the dc bias field, which is oriented along the hard direction for the ordered structure (*y*-axis). For all the reported results both dc and excitation fields are spatially uniform, and the excitation field is a Gaussian pulse applied along *x*-axis, with an amplitude of 1 kA/m and an rms width of 5 ps.

**Figure 4:**
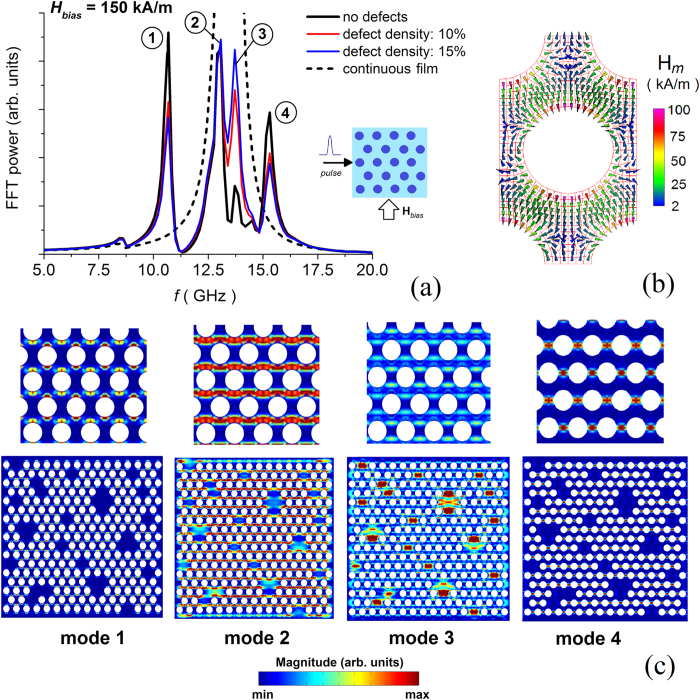
(**a**) FFT power spectra of the average magnetization component parallel to the excitation field (along *x*-axis), calculated for a bias field of 150 kA/m (along *y*-axis): comparison of disordered arrays with different percentages of filled holes to the corresponding ordered one and continuous film. The inset shows external field conditions. (**b**) Spatial distribution of demagnetizing field in the unit cell of the ordered antidot array. (**c**) Surface plots of the magnitude of Fourier coefficients for the FMR modes indicated in (**a**), calculated for the disordered structure with 10% of filled holes (bottom) and relative magnification for the ordered structure (top).

Figure 4a reports the FFT power spectra of the average magnetization component parallel to the excitation field, calculated for a quasi-saturating bias field of 150 kA/m. Disordered arrays with different percentages of filled holes are compared to the corresponding ordered structure and to a continuous film with 8 μm size. The last one exhibits an FFT power spectrum characterized by a broad resonance peak centred at ∼13.5 GHz, while the ordered system displays four well-defined peaks at about 10.7, 13, 13.8 and 15.3 GHz. As demonstrated by the spin wave mode profiles in a magnified area of the non-defective array, the lowest-frequency mode is an edge one (Fig. 4c). The highest spin precession amplitude associated with this mode is found in the hole boundary regions where the demagnetizing field reaches peak values opposed to the bias field^36^,^37^,^38^,^39^ (see the calculated spatial distribution of the demagnetizing field reported in Fig. 4b). The modes at intermediate frequencies are quasi-uniform ones, extending through the whole lattice along stripes orthogonal to the bias field, where the demagnetizing field decreases from ∼25 kA/m (second mode) down to zero (third mode). The final mode is a localized one, with the highest spin precession amplitude localized in between neighbouring holes, where the demagnetizing field changes sign.

As illustrated by Fig. 4a, the insertion of defects produces a decrease in the intensity of the edge and localized modes as well as an enhancement of the quasi-uniform mode at 13.8 GHz. This is well demonstrated by the calculated spin wave mode profiles of the disordered antidot array, reported for the entire sample at the bottom of Fig. 4c. In particular, in the non-patterned regions the spin precession amplitude associated with the third mode increases in a strong way, while becomes negligible for the first and fourth modes. This is due to the uniformity of the demagnetizing field in the interior of the continuous areas and to the related disappearance of magnetic poles. Finally, a negligible shift in the resonance frequencies is found, with the two quasi-uniform modes that tend to the single mode of the continuous film.

A similar behaviour can be found when the quasi-saturating dc bias field is applied along the *x*-axis direction and the Gaussian pulse is directed along *y*-axis (the corresponding FFT power spectra and the associated spin wave mode profiles are reported in Fig. S1 of Supplementary Information).

**Figure 5:**
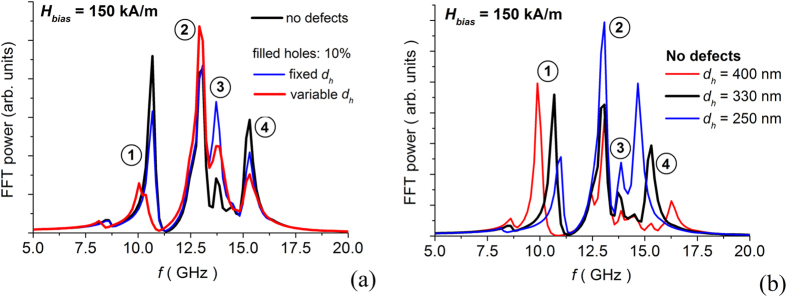
(**a**) FFT power spectra of the average magnetization component parallel to the excitation field (along *x*-axis), calculated for a bias field of 150 kA/m (along *y*-axis): comparison of disordered array with 10% of filled holes and variable hole diameter *d*_*h*_ (red curve) to the corresponding disordered array with 10% of filled holes and *d*_*h*_ = 330 nm (blue curve) and ordered one (black curve). For the antidot array with variable hole diameter, *d*_*h*_ = 280 nm for 20% of non-filled holes, 330 nm for 60% and 380 nm for 20%. (**b**) FFT power spectra of ordered antidot arrays with different hole diameter, ranging from 250 to 400 nm.

Then, we study the combined effects of local variations in the hole size and non-patterned regions. These effects are illustrated by the FFT power spectra reported in Fig. 5a, where the black curve corresponds to the antidot array without defects (hole diameter *d*_*h*_ is fixed to 330 nm); the blue one is associated with the disordered array with 10% of filled holes and *d*_*h*_ set at 330 nm; the red one refers to a disordered array with 10% of filled holes and containing holes with randomly distributed diameter, ranging from 280 nm to 380 nm. In this case, *d*_*h*_ is equal to 280 nm for 20% of non-filled holes, 330 nm for 60% and 380 nm for 20%. When introducing variations in the hole diameter, the behaviour of the system is less predictable, exhibiting a shift in the resonance frequencies, which cannot be controlled and depends in a strong way on the lattice distribution of hole size. This result can be more easily understood by analyzing the FMR behaviour of ordered antidot arrays with holes having fixed diameter ranging from smaller to larger values than 330 nm (Fig. 5b). In particular, if *d*_*h*_ = 250 nm, we observe a narrowing of the frequency interval between the lowest and highest resonance frequencies, with an increase in the intensity of the quasi-uniform and localized modes^13^. Moreover, there is a weakening of the edge mode, due to the contraction of the hole boundary regions characterized by the highest values of the demagnetizing field (see Fig. S2a of Supplementary Information). Conversely, when *d*_*h*_ = 400 nm, the FMR behaviour is spread over a more extended frequency interval with a strong increase in the intensity of the edge mode and a detriment of the others, as a consequence of the highest contribution of the demagnetizing field to magnetization precession. In this case, negligible demagnetizing field values can be found in very limited portions of the sample (see Fig. S2b of Supplementary Information).

**Figure 6:**
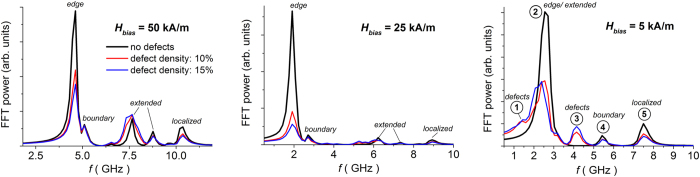
FFT power spectra of the average magnetization component parallel to the excitation field (along *x*-axis), calculated for different amplitudes of bias field (along *y*-axis): comparison of disordered arrays with different percentages of filled holes to the corresponding ordered one.

**Figure 7:**
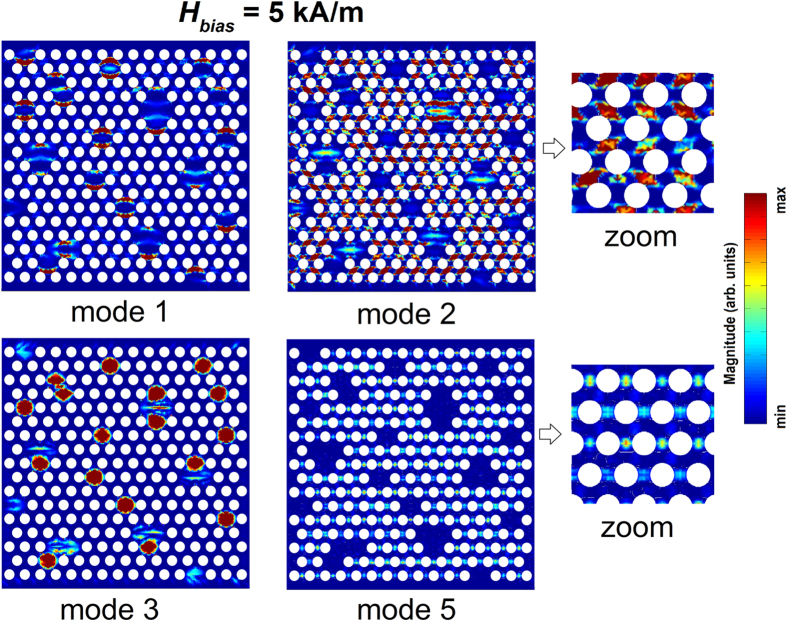
Surface plots of the magnitude of Fourier coefficients for some of the FMR modes obtained when the bias field is equal to 5 kA/m. Left: spin wave mode profiles calculated for a disordered array with 10% of filled holes; right: magnification of the hybridized edge/extended (labelled as 2) and localized (labelled as 5) modes of the corresponding ordered array.

As a final aspect, we investigate the role of the bias field amplitude, by considering again a dc field applied along the *y*-axis. As demonstrated by Fig. 6, which compares disordered arrays with different percentages of filled holes to the corresponding ordered structure, the decrease in the bias field leads to an overall reduction in the resonance frequencies observable in the FFT power spectra^40^. For the non-defective array, there is a relative intensification of the edge mode, which results characterized by a larger precessional amplitude than the quasi-uniform and localized modes. For values of the dc field lower than 20–25 kA/m, a hybridized edge/extended mode^41^,^42^ arises with spatial localization following the magnetization rotation (see the relative spin wave mode profile in a magnified area of the ordered array, reported in Fig. 7). This transition in the system behaviour occurs along the static hysteresis loop when the leaf-type magnetic domain configuration rotates of 30 ° with respect to the *y*-axis, resulting in an abrupt variation in the slope of the curve of magnetostatic energy versus applied field (Fig. S3 in Supplementary Information)^35^.

For a bias field of 5 kA/m, the FFT power spectrum exhibits two distinguishable modes in addition to the hybridized edge/extended one. At 5.5 GHz, there is a mode whose precessional amplitude is larger at the film boundaries, where localized shape anisotropy effects are present; the mode at 7.5 GHz is the localized one (see Fig. 7).

When introducing lattice defects (filled holes), there is again a weakening of the edge and localized modes and, contemporary, the appearance of new modes confined in the non-patterned regions (observed at 1.4 and 4.1 GHz for a 5 kA/m bias field). This is a consequence of the non-synchronous rotation of the magnetization: as discussed before, when the field is applied along the hard direction for the ordered lattice, the magnetization switching in the continuous regions is initially delayed with respect to patterned areas (Fig. 3c,d).

### Frequency-based detection of magnetic beads

Recently, P. J. Metaxas *et al.*^9^ demonstrated that magnetic antidot arrays can be employed for developing high-sensitivity magnetic field sensors, which exploit FMR behaviour in the Gigahertz range. In particular, it was shown that magnetic nanoparticles adsorbed on the surface of a Ni_80_Fe_20_ antidot array can cause a shift in the frequencies of the sample FMR modes. The important result is that the observed shifts, at extended and localized (side) modes for the considered experimental conditions, exceed measurement uncertainty.

Here, we simulate the dynamic response in presence of randomly distributed magnetic microbeads of Ni_80_Fe_20_ antidot arrays with the same geometrical properties of the ordered structure investigated in the previous section. The aim is to compare the estimated shifts in resonance frequencies caused by the bead stray fields to the frequency modifications associated with lattice defects, such as local variations in the hole diameter. The beads, supposed to have similar features of commercial 1 μm Dynal beads, are approximated as magnetic dipoles with saturation magnetic moment of 2.45 × 10^−14 ^Am^2^ and magnetization curve described by Langevin function^43^ (with parameters obtained by fitting the magnetization curve reported in the manufacturer’s Web site^44^). The beads are distributed on the film surface in correspondence of the holes, where the magnetic coupling is higher. In particular, their barycentre is located at a vertical distance of 500 nm from the film plane, at the centres of the holes.

**Figure 8:**
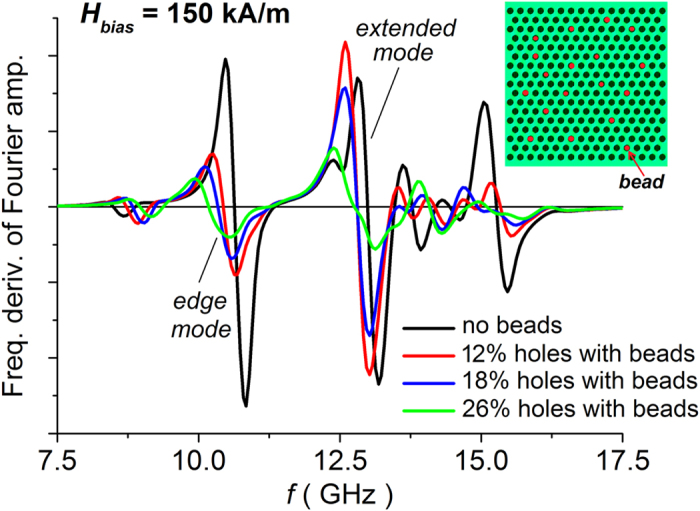
Calculated frequency derivatives of the FFT power spectra (in arbitrary units) of the magnetization component along the excitation field direction (*x*-axis), for a dc bias field of 150 kA/m (applied along *y*-axis). The curves compare the FMR response of an ordered array to the stray fields generated by magnetic microbeads adsorbed on the film surface in different percentages (see scheme in the inset).

The obtained results are reported in Fig. 8, which compares the calculated frequency derivatives of the FFT power spectra for different percentages of beads, considering a bias field of 150 kA/m, applied along *y*-axis, and the same excitation field of the previous section. The magnetic stray fields produced by the beads interact with the magnetization distribution in the antidot array, causing a clear shift in the resonance frequencies of the edge and first extended modes (respectively equal to −0.46 GHz and −0.24 GHz for the case of 26% hole-bead filling). This result confirms the suitability of magnetic antidot arrays for magnetosensing applications that exploit FMR behaviour. However, it highlights the need for an accurate control of lattice order on the long-range scale. As a matter of fact, the estimated shifts in resonance frequencies are lower in magnitude than the one obtained for the edge mode, when considering a disordered structure with a percentage of randomly distributed filled holes of 10% and hole diameter ranging from 280 nm to 380 nm (see Fig. 5a). For this case, a variation of −0.62 GHz is found, due to the localized alteration of the demagnetizing field around the holes with different size.

## Discussion

A micromagnetic numerical approach has been applied to study hysteresis, magnetotransport and FMR properties of Ni_80_Fe_20 _antidot arrays with hexagonal lattice, in the presence of microstructural defects (non-patterned areas and variations in hole diameter). The study has demonstrated that continuous regions can lead to a weakening of the six-fold anisotropy and to a reduced dependence of static behaviour on the applied field orientation, as also confirmed by the experimental hysteresis loops and magnetoresistance curves of an antidot array fabricated by self-assembling polystyrene nanosphere lithography. Moreover, the presence of non-patterned regions lead to a modification of the dynamic response of the system with a softening of the edge and localized modes in the FFT power spectra. This is accompanied by a possible amplification of the extended modes at quasi-saturation fields and to a local alteration of spin wave mode profiles. At low fields, new modes in the continuous regions are observed due to the non-synchronous rotation of the magnetization with respect to patterned areas.

Finally, local variations in the hole diameter can lead to a shift in the resonance frequencies of edge and quasi-uniform modes, impacting on the possible use of antidot arrays in frequency-based sensors for magnetic nanoparticle detection. To avoid alterations of the expected FFT power spectra, more frequent for self-assembling samples, fabrication processes based on EBL techniques can be adopted, enabling to obtain well-defined lattice features, but at the cost of low speed and small covering area.

## Methods

### Sample fabrication

Ni_80_Fe_20_ antidot arrays have been fabricated by means of an optical lithography process that uses polystyrene nanospheres as diffraction masks^45^. In brief, a continuous Ni_80_Fe_20_ thin film with a thickness of 30 nm is prepared by sputtering onto a Si/Si-oxide substrate. An optical photoresist is spun on its top, then a monolayer of polystyrene nanospheres having a diameter of 500 nm is deposited on the resist. Next, the sample is exposed to UV radiation (λ = 350 nm), exploiting the spheres as diffraction masks. After removal of the spheres by sonication and development of the resist, an Ar^+^ sputter etching process is used to remove the magnetic material not protected by the remaining resist. Finally, Ni_80_Fe_20 _holes with a circular shape appear on the surface, giving rise to the antidot array. The centre-to-centre distance of the holes (500 nm) corresponds to the diameter of the spheres, whereas the hole diameter is ∼330 nm.

### Experimental characterization of static magnetic properties

Room-temperature hysteresis loops have been measured by means of a vibrating sample magnetometer (VSM) LakeShore Model 7410 on approximately square samples with a lateral size of 3 mm × 3 mm. The VSM has a computer-controlled rotating head that allows the measurement of hysteresis loops by applying the magnetic field along arbitrary directions in the sample plane. Two orthogonal directions (roughly parallel to the sample edges) have been considered, and labelled *u*- and *v*-axis in Fig. 1d.

Room-temperature magnetoresistance measurements have been performed by means of a standard 4-contacts technique. The electrical contacts cover the whole sample width, to avoid inhomogeneities in the injection of the current^46^. The measurements have been done in the longitudinal and transverse configurations, the magnetic field being in the sample plane in both cases.

### Micromagnetic and magnetotransport modelling

The micromagnetic simulations are performed by means of an advanced solver, which has engineered to run on Graphics Processing Units (GPUs) to accurately reconstruct nanoscale magnetization processes also in large scale samples^31^,^32^. Here, the magnetic antidot array is subdivided into a grid of macrocells (corresponding to the spatial period), in turn discretized with a mesh of hexahedra having size in the order of exchange length (∼5 nm). In each hexahedron, the magnetization vector **M** is assumed to be uniform and is time-updated by integrating the LLG equation:


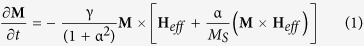


where γ is the absolute value of the gyromagnetic ratio, α is the damping constant, *M*_*S*_ is the saturation magnetization and **H**_*eff*_ is the effective field (sum of the exchange, demagnetizing, anisotropy and applied fields). **H**_*eff*_ can also include the magnetic stray field produced by magnetic nano-/micro-objects (e.g., particles, beads) localized in proximity to the sample surface. In the case studied in the last section, the magnetic moment of the microbead is in turn influenced by the stray field generated by the magnetization distribution in the antidot array^43^.

The exchange field in the LLG equation is evaluated by means of a non-standard finite difference approach able to handle non-structured meshes. This method, based on Taylor series expansion around the computational point, determines second-order derivatives in the exchange term by solving an over-determined set of linear equations^34^. The demagnetizing field **H**_*m*_, locally decomposed into a short- and a long-range term, is efficiently calculated by means of a Fast Multipole technique suitable for large patterned samples^33^. In particular, the short-range term describes the interactions with a low number of macrocells (*N*_*near*_) adjacent to the one containing the element of calculus (centred in **r**_0_). This is evaluated by integrating and summing the Green function gradients over the boundary of each element Ω_*i*_ belonging to the *N*_*near*_ macrocells:


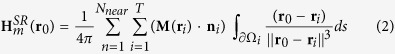


where *N*_*near*_ = (2λ + 1)^2^ and λ is the number of adjacent macrocell layers considered in the approximation.

The long-range term, which describes the interactions with the remaining far macrocells (*N*_*far*_), is evaluated by the multipole expansion technique with order *p*:


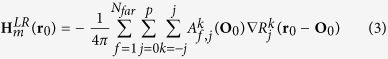


where 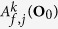 is a time-dependent function that includes the contribution to the cell of calculus (centered in **O**_0_) of all the multipole moments associated with the *N*_*far*_ macrocells. Term 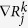 depends only on the spatial discretization and is calculated at the beginning of the simulation.

If the same mesh is adopted for all the macrocells, imposing **M** = 0 in the non-magnetic elements, the time-invariant terms reduce to 6*T*^2^*N*_*near*_ factors for Eq. (2) plus 6(*p *+ 1)^2^*T* factors for the evaluation of multipole moments and the gradient of functions 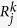.

The calculation of **H**_*m*_ is performed with the lowest multipole expansion order (*p* = 2) and λ = 1, which guarantee a very good accuracy as well as a strong reduction in the number of stored terms and in the computational burden, enabling the treatment of large scale samples^33^.

A norm-conserving scheme based on Cayley transform and Heun algorithm is used for the time-integration of the LLG equation and the efficient determination of equilibrium points, and thus of static hysteresis loops and ground state for the dynamic response^47^,^48^.

Finally, AMR curves are calculated by coupling the micromagnetic solver to a magnetotransport model, which provides the spatial distribution of current density vector at each equilibrium point^49^,^50^. In particular, the electrical conductivity σ(**r**) is described as a spatially dependent function of the local angle θ(**r**) between magnetization and current density vectors, i.e.


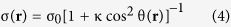


where κ is the AMR ratio and σ_0_ is the electrical conductivity when the material is saturated due to an external field orthogonal to the current flow.

### Simulation parameters

The micromagnetic simulations are performed by considering typical magnetic parameters for Ni_80_Fe_20_, i.e. magnetization saturation is fixed to 800 kA/m, exchange constant to 13 pJ/m and the magnetocrystalline anisotropy is neglected. The magnetorestive curves are calculated by setting the electrical conductivity σ_0_ at 3 MS/m and the AMR ratio at 2%, in agreement with interval ranges reported in the literature^51^,^52^,^53^.

The FMR behaviour is evaluated by setting α at 0.01, considering that for Ni_80_Fe_20_ the damping coefficient is typically in the order of 0.01–0.02 (the corresponding time evolution is calculated by considering a time step of 250 fs). Equilibrium points along static hysteresis loops and magnetoresistive curves and ground state^54^ for the dynamic response are computed by fixing α to 0.1. The increase in α is motivated by the need of reducing the computational time in the determination of equilibrium magnetization configurations^47^. With higher values of α, the convergence to equilibrium points is accelerated and, with the used Cayley transform based time-integration scheme, larger time steps can be introduced without causing stability problems. For the results reported here, equilibrium points have been determined by fixing the time step to 2.5 ps^48^.

## Additional Information

**How to cite this article**: Manzin, A. *et al.* Influence of lattice defects on the ferromagnetic resonance behaviour of 2D magnonic crystals. *Sci. Rep.* **6**, 22004; doi: 10.1038/srep22004 (2016).

## Supplementary Material

Supplementary Information
