# Supplementary material for: Influence of lattice defects on the ferromagnetic resonance behaviour of 2D magnonic crystals
Source: Sci Rep. 2016 Feb 25;6:22004. doi: 10.1038/srep22004 (PMC4766484; doi:10.1038/srep22004)
Supplement: Supplementary Information [file srep22004-s1.pdf]

Supplementary Information

Influence of lattice defects on the ferromagnetic resonance behaviour of 2D magnonic crystals

A. Manzin[1], G. Barrera[1,2], F. Celegato[1], M. Coïsson[1] & P. Tiberto[1]

[1]Istituto Nazionale di Ricerca Metrologica (INRIM), Torino, Italy

[2]Università degli Studi di Torino, Dipartimento di Chimica, Torino, Italy

Corresponding author:

Dr. Alessandra Manzin

Istituto Nazionale di Ricerca Metrologica

Strada delle Cacce, 91

10135 Torino, Italy

tel. +39-011-3919825

e-mail: a.manzin@inrim.it

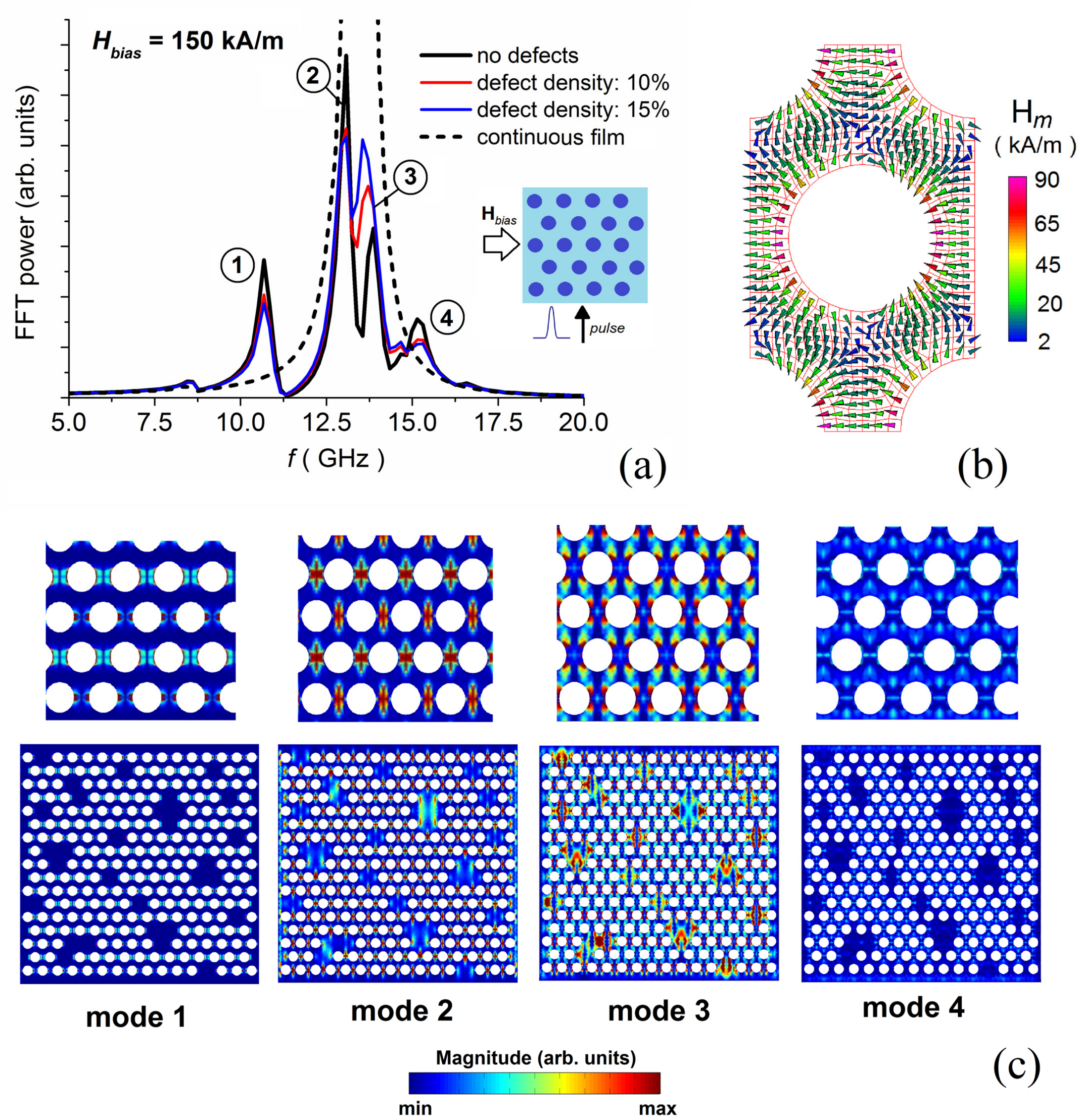

Fig. S1 (a) FFT power spectra of the average magnetization component parallel to the excitation field (*y*-axis), calculated for a bias field of 150 kA/m oriented along *x*-axis: comparison of disordered arrays with different percentages of filled holes to the corresponding ordered one and continuous film. The inset shows external field conditions. (b) Spatial distribution of demagnetizing field in the unit cell of the ordered antidot array. (c) Surface plots of the magnitude of Fourier coefficients for the FMR modes indicated in (a), calculated for the disordered structure with 10% of filled holes (bottom) and relative magnification for the ordered structure (top).

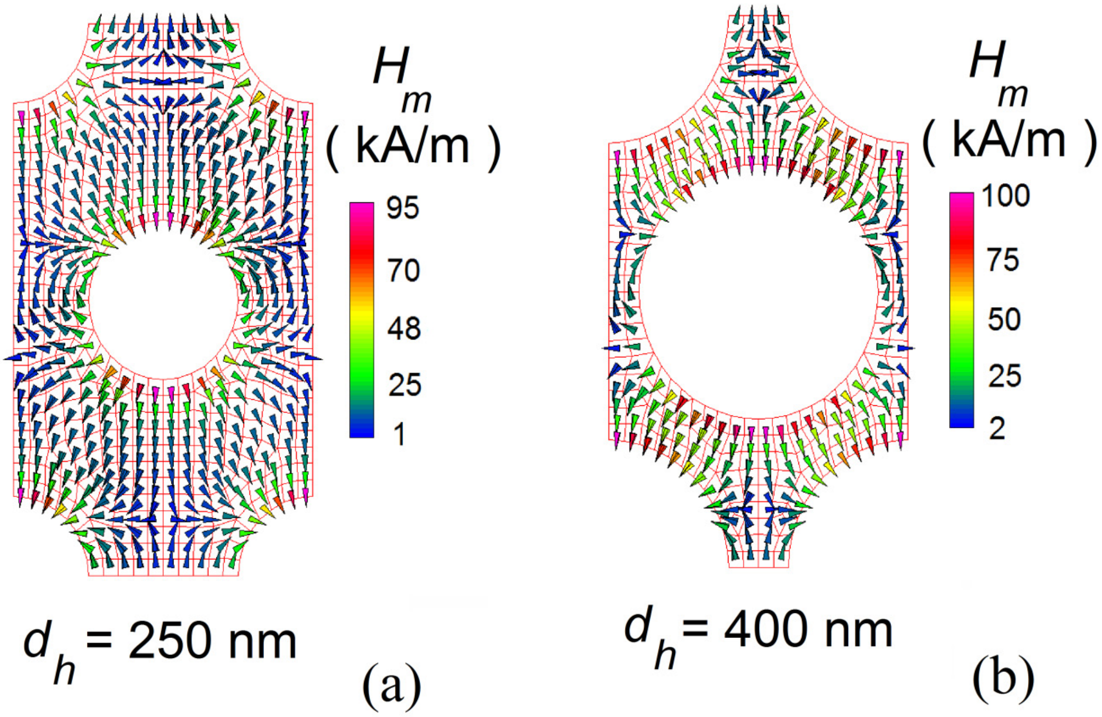

Fig. S2 Calculated spatial distributions of the demagnetizing field in the unit cell of ordered antidot arrays with hole diameter d_h equal to (a) 250 nm and (b) 400 nm. The corresponding magnetization configuration (quasi-saturation) has been determined by applying a dc field of 150 kA/m along the y-axis direction.

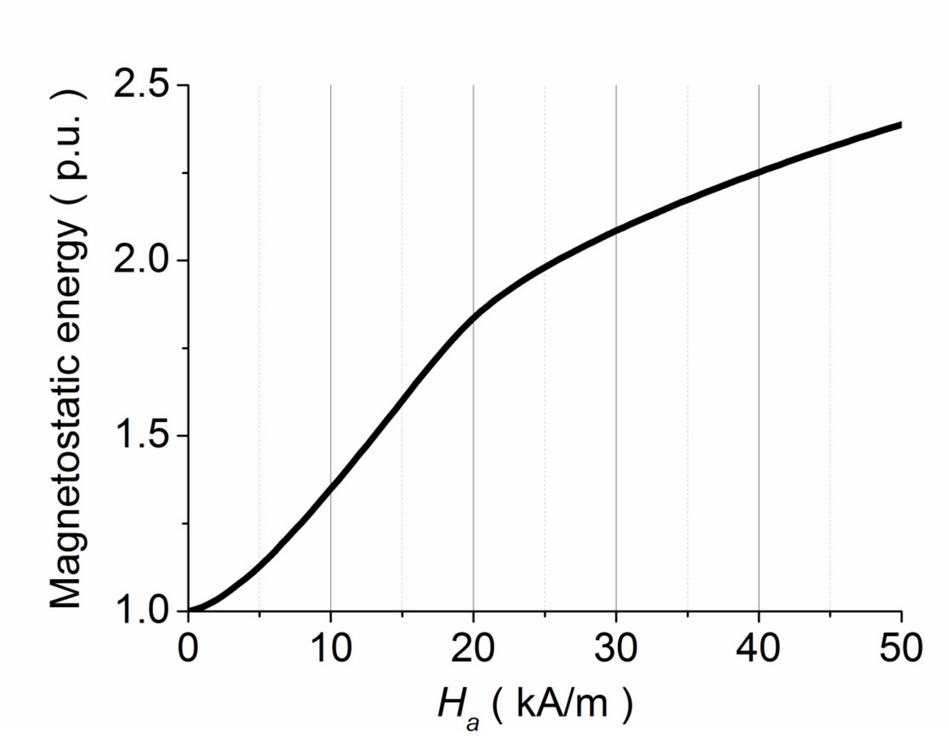

Fig. S3 Evolution of the magnetostatic energy versus applied field associated with the *y*-axis static hysteresis loop of the ordered antidot array, reported in Fig. 3b of the manuscript.
